# Evaluation of hematocrit-adjusted conversion strategies for mycophenolic acid and tacrolimus monitoring using volumetric absorptive microsampling in lung and renal transplant recipients

**DOI:** 10.3389/jpps.2026.16123

**Published:** 2026-03-26

**Authors:** Liang Juan, Chih-Ning Cheng, Wen-Chi Chang, Huai-Hsuan Chiu, Chao-Wen Lu, Hsao-Hsun Hsu, Ching-Hua Kuo, Yu-Ju Tseng

**Affiliations:** 1 School of Pharmacy, College of Medicine, National Taiwan University, Taipei, Taiwan; 2 Faculty of Medicine, Graduate School of Medicine, Kyoto University, Kyoto, Japan; 3 Department of Medical Research, National Taiwan University Hospital, Taipei, Taiwan; 4 Department of Surgery, National Taiwan University Hospital and College of Medicine, National Taiwan University, Taipei, Taiwan; 5 Department of Surgery, National Taiwan University Cancer Center, Taipei, Taiwan; 6 Department of Pharmacy, National Taiwan University Hospital, Taipei, Taiwan

**Keywords:** mycophenolic acid, tacrolimus, therapeutic drug monitoring, transplantation, volumetric absorptive microsampling

## Abstract

**Objectives:**

Mycophenolic acid (MPA) and tacrolimus (TAC) exhibit substantial pharmacokinetic variability, and volumetric absorptive microsampling (VAMS) offers a minimally invasive alternative for therapeutic drug monitoring (TDM). This study aimed to develop a VAMS-based method for MPA and TAC quantifications and systematically evaluate hematocrit (Hct)-adjusted conversion strategies.

**Methods:**

Adult transplant recipients receiving mycophenolate mofetil or mycophenolate sodium were prospectively enrolled. Paired plasma (MPA), whole-blood (TAC), and VAMS samples were analyzed using validated LC–MS/MS method for simultaneous MPA and TAC quantification. Multiple Hct-adjusted conversion approaches for MPA were compared using Passing-Bablok regression, Bland-Altman analysis, and predictive performance metrics. Clinical applicability was assessed through scenario-based AUC estimation MPA AUC estimations under different sampling schemes.

**Results:**

LC-MS/MS method for quantifying MPA and TAC in VAMS exhibited good linearity (R^2^ > 0.99) and accuracy within 85–115% across validation ranges (10–20,000 ng/mL for MPA; 0.5–500 ng/mL for TAC). Formula A-ind [(VAMS/1 – individual Hct) x f_bpp_], where f_bpp_ represents the MPA protein binding fraction (0.97), achieved clinical agreement in 86% of samples for the conversion of MPA concentrations between VAMS and plasma, representing the most balanced between predictive reliability and operational feasibility. TAC concentrations from VAMS correlated strongly with whole blood values without requiring Hct correction. Clinical case applications showed that rich eight-point VAMS sampling enabled more accurate AUC estimations than conventional three-point schemes, further highlighting the advantages of using VAMS for MPA TDM.

**Conclusion:**

This validated VAMS-based approach offers a minimally invasive and clinically applicable alternative to venous sampling for MPA and TAC monitoring. Incorporating individualized Hct adjustments improves predictive performance, supporting conditional integration of VAMS into routine TDM.

## Introduction

Organ transplantation is one of the greatest achievements in modern medicine, providing life-saving therapy for patients with end-stage organ failure. In 2023, over 46,000 organ transplants were performed in the United States, and Taiwan has similarly shown a steady annual increase of 400–500 cases [1, 2]. Long-term graft survival relies on appropriate immunosuppression, most commonly based on a combination regimen of tacrolimus (TAC) with mycophenolate mofetil (MMF) or mycophenolate sodium (MPS), often together with corticosteroids.

TAC, a calcineurin inhibitor, suppresses T-cell activation but is characterized by a narrow therapeutic index and marked pharmacokinetic (PK) variability [3]. Underexposure increases the risk of acute rejection, whereas overexposure has been associated with nephrotoxicity, neurotoxicity, metabolic complications, and infection. Consequently, routine therapeutic drug monitoring (TDM) of TAC trough concentrations in whole blood is standard practice in transplant care [4, 5]. TAC is extensively distributed into erythrocytes, and whole blood is therefore the recommended matrix for monitoring.

In contrast, optimizing exposure assessment for mycophenolic acid (MPA) remains more challenging in clinical practice. MMF and MPS are rapidly converted after oral administration to MPA, an inosine monophosphate dehydrogenase inhibitor that suppresses lymphocyte proliferation. MPA is predominantly confined to the plasma compartment and exhibits high plasma protein binding (∼97%), with minimal red blood cell partitioning. Similar to TAC, MPA displays substantial inter- and intra-individual PK variability, influenced by enterohepatic recirculation, albumin concentration, and metabolic pathways. Both inadequate exposure and excessive toxicity—such as gastrointestinal intolerance, bone marrow suppression, and infection—may necessitate dose adjustment or discontinuation [6, 7]. However, unlike TAC—where trough-based monitoring is well standardized—MPA exposure assessment often requires multi–time-point sampling to estimate area under the concentration–time curve (AUC), creating practical challenges in routine clinical settings.

Because both inadequate exposure and excessive toxicity are concerns in MMF/MPS therapy, the relationship between MPA concentrations and outcomes has been extensively investigated [8–11]. Increasing evidence supports the need for TDM, especially in patients at high risk of rejection or toxicity [12–14]. Currently, limited sampling strategies (LSS) utilizing AUC are suggested to optimize therapy, yet their effectiveness and clinical feasibility remain inconsistent. In particular, AUC estimation requires multiple timed samples, increasing procedural burden and cost [15, 16].

Dried blood spot (DBS) and volumetric absorptive microsampling (VAMS) techniques have emerged as convenient alternatives for fingertip blood collection. Several reviews and expert consensus reports have summarized the analytical performance, clinical applicability, and practical considerations of DBS/VAMS-based TDM for immunosuppressants, including MPA and TAC [17–20]. Compared with venous sampling, these approaches require minimal training, generate less waste, and simplify storage and transport—making them suitable for frequent, decentralized, or home-based monitoring [21]. Nonetheless, systematic reviews highlight ongoing barriers to routine implementation, including suboptimal accuracy, variability in patient self-collection, and issues with sample stability and drying [18, 22–24]. To address these challenges, standardized, professionally supervised procedures must first be validated before extending use to home-based TDM.

Despite its potential, microsampling-based TDM for MPA poses additional challenges. MPA predominantly resides in the plasma fraction and is highly protein-bound (∼97%), making whole blood measurements non-equivalent to plasma concentrations [25–28]. Because most existing MPA TDM data and therapeutic targets are derived from plasma samples, establishing accurate conversion from VAMS whole blood to plasma concentrations is essential for clinical interpretation. Furthermore, variations in hematocrit (Hct) alter blood viscosity and analyte distribution, affecting quantification accuracy. Reliable Hct conversion strategies—through empirical regression, correction formulas, or population-based models—are therefore crucial; however, systematic comparisons and standardization remain limited [29–33].

Therefore, this study aimed to develop and validate a VAMS-based approach as a practical alternative to venous sampling for MPA TDM. By systematically comparing multiple conversion strategies and assessing analytical performance, we sought to establish a conversion method that is not only accurate but also feasible for real-world clinical application. To align with real-world transplant practice, TAC was concurrently analyzed, as both drugs are commonly co-administered in transplant recipients. Simultaneous VAMS-based monitoring of MPA and TAC may streamline clinical workflows and facilitate future expansion toward remote or home-based TDM [17, 19].

## Materials and methods

### Study design

This was a prospective study with sequential purposive sampling conducted at National Taiwan University Hospital and approved by the institutional Research Ethics Committee (202302126RINA). Adult patients (≥18 years) receiving MMF (CellCept®) or enteric-coated MPS (Myfortic®) for more than 3 days were eligible. Participants receiving TAC as part of their immunosuppressive regimen were treated with either immediate-release TAC (Prograf®) or prolonged-release TAC (Advagraf®), both of which were included in the study cohort. All participants provided written informed consent. To enhance clinical relevance, we enriched the cohort by design with patients for whom MPA monitoring had been recommended by clinical specialists because of their medical condition, suspected adverse effects, or immunosuppressive regimen. This approach included individuals at higher risk of drug-related complications and more accurately reflected the intended target population for VAMS-based TDM.

### Sample collection and preparation

Blood samples were collected by trained nurses between June and October 2023. After MMF or MPS administration, whole blood was drawn from each patient; part of the sample was used to prepare VAMS specimens (20-µL Mitra® Tips, Neoteryx, Torrance, CA, USA), and the remainder was centrifuged to obtain plasma. Paired VAMS, plasma, and whole-blood samples were used for subsequent analyses similar to that of previous studies [27, 34, 35]. VAMS devices were dried in the dark at room temperature for at least 2 h, stored in double-layer zip bags with desiccants, and kept at −20 °C until analysis.

For sample preparation, VAMS tips were incubated with 200 µL deionized water and mixed at 1,000 rpm for 10 min (Geno/Grinder 2010, SPEX Sample Prep, Metuchen, NJ). Subsequently, 185 µL methanol containing internal standards (final concentrations: 200 ng/mL mycophenolate-d_3_ and 40 ng/mL tacrolimus-^13^C-d_2_) and 10 µL 0.1 M zinc sulfate were added, followed by an additional 10 min of mixing at 1,000 rpm. Whole-blood samples underwent the same extraction and centrifugation steps as VAMS but without the initial deionized water. Plasma samples were extracted with 150 µL methanol containing the same internal standards and processed under identical mixing and centrifugation conditions. All samples were centrifuged at 15,000 g for 10 min, and supernatants were filtered through 0.22-µm RC-4 filters (Sartorius, Göttingen, Germany) before LC–MS/MS analysis.

MPA and TAC working solutions, together with their isotope-labeled internal standards (MPA-d_3_ and TAC-^13^C-d_2_), were prepared in methanol by serial dilution of stock solutions and stored at −20 °C. Calibration ranges were 10–20,000 ng/mL for MPA (10, 50, 100, 200, 500, 1,000, 2,000, 10,000, and 20,000 ng/mL) and 0.5–500 ng/mL for TAC (0.5, 1.0, 2.5, 5.0, 10, 25, 50, 100, and 500 ng/mL). For method validation, calibration curves were prepared in plasma and whole-blood matrices by plotting analyte-to-internal-standard peak area ratios and fitted using a weighted (1/x) quadratic regression model, with correlation coefficients (R^2^) consistently >0.99.

The lower limits of quantification (LLOQ) were 10 ng/mL for MPA and 0.5 ng/mL for TAC. Low, medium, and high quality-control (QC) samples were prepared at 20, 200, and 2,000 ng/mL for MPA and 1, 10, and 100 ng/mL for TAC, respectively. Limits of detection and quantification were defined by signal-to-noise ratios >3 and >10. Intra-day precision and accuracy were assessed using triplicate QC samples at each level for all matrices, including VAMS. Accuracy was required to be within 85–115% (80–120% for LLOQ), and precision criteria were coefficients of variation (CV) ≤15% for QC levels and ≤20% for the LLOQ. Recoveries were determined by comparing peak area ratios of analytes spiked into blank plasma or whole blood before extraction (pre-spiked) with those spiked after extraction (post-spiked). Matrix effects were evaluated by comparing peak area ratios of post-spiked plasma with those of aqueous standards. Values close to 100% indicated negligible matrix effects, and deviations within ±20% were considered acceptable [36].

### LC-MS/MS methods for analyzing MPA and TAC

Chromatographic separation was performed on an Agilent 1290 UHPLC system coupled to an Agilent 6460 triple-quadrupole mass spectrometer (Agilent Technologies, Waldbronn, Germany) using an Acquity C18 column (1.7 µm, 2.1 × 100 mm; Waters, Milford, MA, USA). The mobile phases consisted of 2 mM ammonium acetate with 0.1% formic acid in water (A) and acetonitrile/isopropanol (4:6, v/v) (B). A gradient was applied from 50% to 100% B over 3 min, held until 4.5 min, and returned to 50% B by 5.5 min at a flow rate of 0.3 mL/min. Processed samples were injected via autosampler. The ion source was operated in multiple-reaction monitoring (MRM) mode with standard operating conditions (ion spray voltage 4,000 V, sheath gas 40 psi at 325 °C, nitrogen as collision gas). MRM transitions for each analyte and internal standard are summarized in Supplementary Table S1.

### Comparisons of different conversion strategies

Four previously described approaches for Hct-related conversion were evaluated: two derived from DBS sampling and two from VAMS sampling [29, 30, 32, 33]. For the present analysis, all equations were applied using VAMS concentrations in place of DBS concentrations. Wenkui et al. and Kromdijk et al. proposed similar DBS-based equations, with Kromdijk et al. recommending the use of a fixed mean Hct of 0.45 L/L to facilitate clinical application [30, 33]. In this study, this model was evaluated in two variations: Formula A-fixed (A-fix), applying a constant Hct = 0.45 L/L for all patients, and Formula A-individualized (A-ind), substituting each patient’s measured Hct value to allow individualized conversion.

$$Formula\;A:\left[\frac{{VAMS}_{\left[analyte\right]}}{1-Hct}\right]\times f_{bpp}={plasma}_{analyte}$$

Zimmermann et al. developed a VAMS-specific linear model with Hct normalized to 0.45(Formula B) [32].

$$Formula\;B:C_{Plasma}=A\cdot C_{VAMS}+B\cdot \frac{Hct}{0.45}+C\cdot \frac{Hct}{0.45}\cdot C_{VAMS+Intercept}$$

In addition, we derived a multiple linear regression model using VAMS concentration and individual Hct as predictors (Formula C).

$$Formula\;C:C_{Plasma}=A\cdot C_{VAMS}+B\cdot Hct+Intercept$$

Finally, based on the approach described by Jager et al., we applied a DBS-derived conversion using the mean Hct of our cohort and the blood cell-to-serum partitioning constant K_BC:Serum_ (Formula D) [29].

$$Formula\;D:\frac{{Analyte}_{VAMS}}{\left(1-Hct\right)+K_{BC:Serum}\cdot Hct}={Analyte}_{serum}$$

### Statistical analysis

Patient demographics were summarized using descriptive statistics. To evaluate whether VAMS-derived concentrations could be used interchangeably with conventional plasma measurements and to identify the most appropriate conversion strategy, a stepwise analytical framework was applied.

Systematic bias between VAMS-derived and plasma concentrations was first assessed using Passing–Bablok regression, which estimates proportional and constant bias. Conversion formulas with regression slopes and intercepts whose 95% confidence intervals included 1 and 0, respectively, were considered to exhibit minimal systematic bias. In parallel, median percentage predictive error (MPPE) was calculated to quantify overall bias of back-calculated plasma concentrations. Dispersion of prediction errors was evaluated using median absolute percentage error (MAPE), with values <15% considered acceptable, providing a complementary measure of the magnitude of deviation between predicted and observed concentrations. Analytical agreement was subsequently assessed using Bland–Altman analysis. Predefined agreement criteria required a mean relative bias within ±20%, and the proportion of samples falling within these limits was calculated to summarize overall agreement [37]. To further differentiate the performance among candidate conversion formulas, a stricter agreement threshold of ±10% relative difference was applied in an exploratory analysis. To identify factors associated with failure to meet this stricter criterion, logistic regression analysis was performed. When a significant association was identified, receiver operating characteristic (ROC) curve analysis was conducted to assess discriminative ability and to determine an optimal threshold using Youden’s index. Clinical agreement was evaluated separately using predefined thresholds: an absolute difference of ≤100 ng/mL for concentrations <1,000 ng/mL and a relative difference of ≤10% for concentrations ≥1,000 ng/mL. The final Hct conversion method was selected based on the combined evidence from bias assessment, dispersion metrics, analytical agreement, clinical agreement, and practical applicability.

To explore the clinical implications, MPA AUC was calculated using the trapezoidal rule under several sampling scenarios: inpatient VAMS with intensive sampling (pre-dose, 0.5, 1, 2, 4, 6, 8, and 12 h), inpatient sampling at C_0_, C_2_, and C_12_, and outpatient sampling at C_0_, C_2_, and C_6_, thereby illustrating how multi-point VAMS sampling may support accurate AUC estimation for evaluating adverse effects. All statistical analyses were performed using RStudio 4.4.2 (Boston, MA) and Python 3.10.

## Results

### MPA and TAC VAMS method establishment and validation

With the optimized VAMS sample preparation and LC-MS/MS setting, MPA and TAC were eluted at 1.24 min and 2.84 min, respectively, with proper separation between the analytes. The optimized MRM chromatogram of both analytes is provided in Supplementary Figure S1. Method validation showed good linearity, with ranges of 10–20,000 ng/mL for MPA and 0.5–500 ng/mL for TAC, and both calibration curves yielding R^2^ > 0.99 (Supplementary Table S2). Intra-day accuracy and precision were assessed using four concentration levels of quality controls. VAMS MPA showed accuracy of 83.36–99.16% and precision of 1.60–4.48%, while TAC showed accuracy of 85.61–89.37% and precision of 2.07–7.68% (Table 1). Extraction recovery was relatively consistent across QC concentrations, ranging from 79.92 to 91.02% for MPA and 98.85–114.11% for TAC. The matrix effect ranged from 94.98% to 111.52% for MPA and 95.30%–104.99% for TAC after internal standard correction, indicating that MPA-d_3_ effectively corrected for matrix effects in VAMS samples. Taken together, these validation results support the analytical reliability of the established VAMS method.

**TABLE 1:** Intra-day accuracy and precision, matrix effect, and recovery of VAMS MPA and TAC at different concentration levels.

Analyte	Levels	Intra-day (n = 3)		Extraction recovery		Matrix effect			
						Without ISTD		ISTD	
		Accuracy (%)	Precision (RSD%)	Recovery (%)	Precision (RSD%)	Matrix effect (%)	Precision (RSD%)	Matrix effect (%)	Precision (RSD%)
MPA^a^	LLOQ	83.36 ± 2.40	2.88	79.92 ± 2.89	3.61	43.23 ± 2.65	2.88	105.70 ± 8.31	7.87
	LQC	89.01 ± 3.27	3.68	86.23 ± 3.62	4.20	41.31 ± 1.10	3.68	111.52 ± 10.53	9.44
	MQC	90.29 ± 4.05	4.48	81.26 ± 0.25	0.30	42.19 ± 0.14	4.48	103.68 ± 3.61	3.48
	HQC	99.16 ± 1.59	1.60	91.02 ± 0.29	0.32	63.85 ± 0.29	1.60	94.98 ± 2.92	3.07
TAC^b^	LLOQ	85.61 ± 6.57	7.68	99.71 ± 3.23	3.24	101.89 ± 3.29	7.68	103.02 ± 6.98	6.78
	LQC	89.37 ± 4.03	4.51	114.11 ± 7.70	6.75	83.66 ± 7.71	4.51	104.99 ± 2.99	2.85
	MQC	88.97 ± 2.79	3.14	100.10 ± 0.66	0.66	99.88 ± 1.25	3.14	98.84 ± 2.03	2.06
	HQC	89.04 ± 2.07	2.07	98.85 ± 1.65	1.67	103.50 ± 0.98	2.07	95.30 ± 1.21	1.27

HQC, higher quality control; ISTD, internal standard; LLOQ, lower limit of quantification; LQC, lower quality control; MPA, mycophenolic acid; MQC, middle quality control; RSD, relative standard deviation; TAC, tacrolimus; VAMS, volumetric absorptive microsampling.

^a^ LLOQ and LQC, MQC, and HQC concentrations of samples for MPA were 10, 20, 200, and 2,000 ng/mL respectively.

^b^ LLOQ and LQC, MQC, and HQC concentrations of samples for TAC were 0.5, 1, 10, and 100 ng/mL respectively.

### Evaluation of hematocrit conversion strategies for MPA and method comparison for TAC

Twenty-one paired samples were collected: VAMS–plasma for MPA and VAMS–whole-blood for TAC. All samples were quantified using validated methods (Supplementary Tables S2, S3), and corresponding clinical characteristics were provided in Supplementary Table S4. For MPA, four previously described Hct correction approaches (Formulas A–D, see Methods) were compared, including both fixed and individualized variations of Formula A. Each conversion model was evaluated using Passing–Bablok regression, Bland–Altman plots, and predictive performance metrics.

**FIGURE 1:**
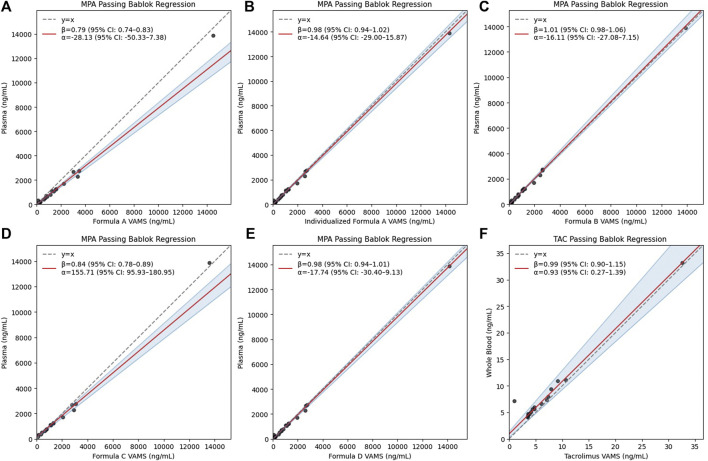
Passing–Bablok regression plots comparing VAMS-derived concentrations from different formulas with conventional reference measurements. Panels are presented in the following order: **(A)** MPA Formula A-fix, **(B)** MPA Formula A-ind, **(C)** MPA Formula B, **(D)** MPA Formula C, **(E)** MPA Formula D, and **(F)** TAC comparison. The dashed gray line represents the line of identity (y = x). The red solid line indicates the Passing–Bablok regression line, with shaded areas representing the 95% confidence intervals. The slope (β) and intercept (α), with corresponding 95% confidence intervals, are displayed in each panel.

**FIGURE 2:**
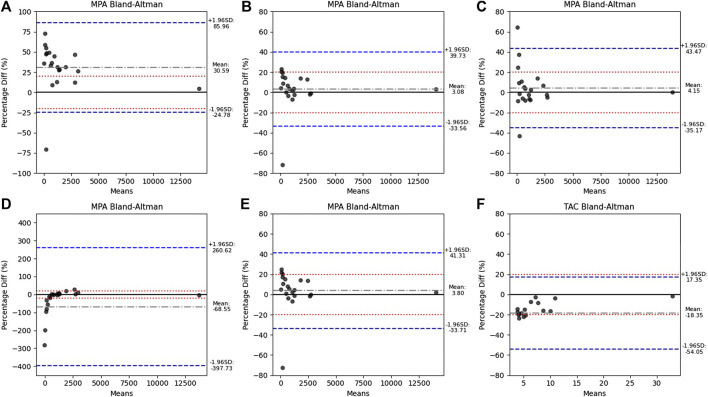
Bland–Altman plots assessing agreement between VAMS-derived concentrations from different formulas with conventional reference measurements. Panels are shown in the following order: **(A)** MPA Formula A-fix, **(B)** MPA Formula A-ind, **(C)** MPA Formula B, **(D)** MPA Formula C, **(E)** MPA Formula D, and **(F)** TAC comparison. The y-axis represents the percentage difference between VAMS and reference measurements. The solid black line indicates zero bias, while the red dotted lines represent±20% agreement. The gray dotted line represents the mean percentage difference (systematic bias), and the blue dashed lines denote the limits of agreement (mean ±1.96 standard deviations). Each point corresponds to one paired sample.

Distinct performance patterns were observed (Table 2). Based on Passing–Bablok regression (Figure 1) and MPPE values, Formula A-ind, Formula B, and Formula D showed the smallest proportional and constant bias, whereas Formula A-fix and Formula C exhibited marked deviation. These three models also yielded lower MAPE values, supporting their superior analytical consistency. Using predefined agreement limits of ±20% in Bland–Altman plots, more than 80% of samples for Formula A-ind, B, and D met the acceptance criteria, indicating acceptable analytical agreement. To further differentiate the performance among these three formulas, a stricter agreement criterion of ±10% was applied. Under this definition, the proportions of samples meeting the criterion were substantially lower, ranging from 57% to 71%. Greater deviations were predominantly observed at lower concentration ranges (Figure 2).

**TABLE 2:** Comparisons of biases between measured and predicted plasma MPA concentrations calculated by different conversion formulas.

Statistics					
Analysis	Formula A (Hct = 0.45)	Formula A (individual Hct)	Formula B	Formula C	Formula D
Passing-bablok regression analysis					
Formula	$C_{VAMS}=0.79x-28.13$	$C_{VAMS}=0.98x-14.64$	$C_{VAMS}=1.01x-16.11$	$C_{VAMS}=0.84x+180.95$	$C_{VAMS}=0.98x+9.13$
Slope	0.79 (0.74–0.83)	0.98 (0.94–1.02)	1.01 (0.98–1.06)	0.84 (0.78–0.89)	0.98 (0.94–1.01)
Intercept	−28.13 (−50.33–7.38)	−14.64 (−29.00–15.87)	−16.11 (−27.08–7.15)	155.71 (95.93–180.95)	−17.74 (−30.40–9.13)
Bland-altman analysis					
% Mean difference (−1.96SD; +1.96SD)	30.59% (−24.78–85.96%)	3.08% (−33.56–39.73%)	4.15% (−35.17–43.47%)	−68.55% (−397.33–260.62%)	3.80% (−33.71–41.31%)
Of samples within±20%	19%	86%	81%	57%	81%
% Of samples within±10%	10%	62%	71%	43%	57%
Predictive performance assessments					
MPPE [%]	33.57	3.73	−0.08	−2.12	5.13
MAPE [%]	35.54	6.93	6.77	11.83	6.56
% Of samples fulfilled clinical requirements^a^	33%	86%	90%	57%	86%

Hct, hematocrit; SD, standard deviation; MPPE, median prediction percentage error; MAPE, median absolute percentage error.

^a^ <100 ng/mL for concentrations below 1,000 ng/mL and <10% for higher concentrations.

To evaluate whether concentrations influenced conversion success under the ±10% criterion, logistic regression analysis was performed, with conversion success as the dependent variable and log-transformed plasma MPA concentration as the predictor. Plasma concentration was identified as a significant determinant (odds ratio 4.41; *p* < 0.05), indicating that higher concentrations were substantially more likely to meet the analytical agreement. This concentration-dependent pattern was further supported by a supplemental ROC analysis (AUC = 0.73; sensitivity 0.84; specificity 0.76; *p* = 0.0008), which identified 488.23 ng/mL as the optimal threshold (Supplementary Figure S2).

When evaluated against clinical thresholds (Table 2), Formula B achieved the highest agreement (90%), followed by A-ind and D (86%). Despite Formula B showing slightly higher numerical agreement, Formula A-ind exhibited smaller bias dispersion (Figure 2) and required simpler calculations, suggesting better practical applicability. Overall, A-ind was selected as the most suitable method for subsequent analyses, balancing analytical robustness with clinical feasibility.

For TAC, Passing–Bablok and Bland–Altman analyses demonstrated good agreement between VAMS and whole-blood concentrations, with a regression slope close to unity and a modest negative bias (mean percent difference = −18.4%, Figure 2F). This bias remained within the predefined acceptance limits, indicating that TAC can be reliably measured by VAMS without requiring Hct correction.

### Clinical applications

**FIGURE 3:**
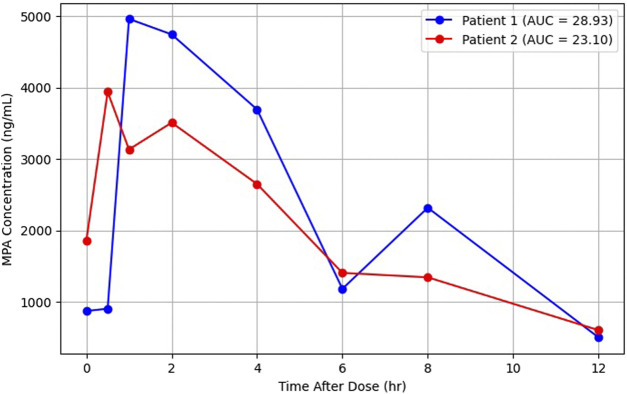
Concentration–time profiles of MPA in two representative patients over a 12-hour dosing interval. Concentrations shown on the y-axis were derived from VAMS using Formula A-ind. Serial samples were collected following oral dosing, and the AUC_0_–_12_ was calculated for each patient (Patient 1: 28.93 mg·h/L; Patient 2: 23.10 mg·h/L), demonstrating interindividual pharmacokinetic variability.

To illustrate clinical applicability, the validated VAMS method was applied to two lung-transplant recipients receiving MMF + TAC who developed leukopenia. Using eight-point VAMS sampling, plasma MPA concentrations were estimated via the A-ind conversion, enabling full AUC profiling (Figure 3; Table 3). The complete eight-point sampling produced the highest AUCs (28.9 and 23.1 mg·h/L), whereas inpatient (C_0_, C_2_, C_12_) and outpatient (C_0_, C_2_, C_6_) schemes progressively underestimated exposure (−16 to −35%). This underestimation highlights the impact of sampling strategy on exposure assessment. TAC troughs from VAMS were consistent with routine whole-blood values. These findings demonstrate that VAMS facilitates minimally invasive assessment of full AUC through multi-time-point sampling and provides a practical platform for the subsequent development and evaluation of LSS.

**TABLE 3:** Summary of clinical characteristics and AUC estimations in two lung transplant patients.

Characteristics	Patient 1	Patient 2
Age (y/o)	41	25
Gender	Female	Female
Diagnosis	LAM, lung Tx	iPAH, HFpEF, lung Tx
Alb (g/dL)	3.6	3.4
Hct (%)	28.5	25.6
MMF dosing	750 mg BID (tapered)	750 mg BID (stopped after day 9)
WBC nadir (K/mcL)	4.87 (day 85)	2.93 (day 15)
AUC estimations (mg·hr/L)		
Full 8-points^a^	28.93	23.10
Inpatient 3-points^b^	24.26	21.59
Outpatient 3-points^c^	18.66	18.90
TAC WB trough (ng/mL)	6.0	7.4
TAC VAMS trough (ng/mL)	5.94	6.95
Comedications	Sirolimus, prednisolone, valganciclovir	Prednisolone, valganciclovir, cefepime

Alb, albumin; AUC, area under the curve; BID, twice a day; Hct, hematocrit; HFpEF, heart failure with preserved ejection fraction; iPAH, idiopathic pulmonary arterial hypertension; LAM, lymphangioleiomyomatosis; MMF, mycopohenolate mofetil; TAC, tacrolimus; Tx, transplantation; VAMS, volumetric absorptive microsampling; WB, whole blood; WBC, white blood cell count.

^a^ Sampling time = 0, 0.5, 1, 2, 4, 6, 8, 12 h after dose.

^b^ Sampling time = 0, 2, 12 h after dose.

^c^ Sampling time = 0, 2, 6 h after dose.

## Discussion

The present study expands upon previous work by establishing a professionally supervised VAMS–LC–MS/MS method for simultaneous quantification of MPA and TAC that meets current bioanalytical validation standards. Although prior studies have demonstrated the feasibility of VAMS for immunosuppressant monitoring, most focused on single analytes and did not integrate systematic evaluation of Hct-adjusted conversion strategies [17, 24, 27, 28, 32]. By directly comparing multiple conversion models under controlled sampling conditions, our study addresses an important gap and provides a clinically oriented assessment of VAMS applicability in transplant pharmacotherapy.

Although the original matrix effects were suboptimal, the use of isotope-labeled internal standards effectively corrected the matrix effects and enabled us to achieve acceptable accuracy and precision. Buchwald et al. reported similar results in a MPA LC–MS/MS assay showing that isotope-labeled internal standards effectively compensate for matrix effects compared with structural analogues, supporting their use in bioanalytical quantitation [38].

Our findings demonstrate that Hct exerts a substantial influence on MPA quantification from VAMS samples. Among the evaluated strategies, the individualized Hct-adjusted correction (Formula A-ind) and the physiologically derived population-based model (Formula D) showed better agreement with plasma concentrations compared with the fixed-Hct approach (Formula A-fix) and the simpler regression model (Formula C). Although Formula C incorporates Hct as a covariate, it lacks an interaction term between Hct and concentration and therefore functions as a partially adjusted rather than a fully Hct-corrected model, resulting in greater proportional bias across the concentration range. Formula B, which includes both Hct and a concentration–Hct interaction term, yielded slightly superior predictive statistics but at the cost of increased model complexity, limiting its practicality for routine clinical use. Taken together, Formula A-ind represents the most balanced compromise between predictive reliability, interpretability, and operational feasibility for clinical application.

Beyond the choice of conversion model, achieving agreement within a stringent ±10% threshold may be constrained by residual biological and clinical variability. Variations in plasma protein binding (albumin, α_1_-acid glycoprotein, bilirubin) and clinical heterogeneity—including post-transplant recovery stage, inflammatory burden, and concurrent immunosuppressive therapy—may further influence individual PKs and limit the precision achievable by any single correction approach [6, 7, 39]. From a clinical perspective, based on the predefined clinical agreement criteria, the acceptance rates observed in our study were generally comparable to—and in several instances higher than—those reported in previous VAMS studies [10, 12, 21, 23]. In addition, ROC analysis demonstrated that conversion accuracy declined mainly at very low concentrations, with a threshold of approximately 488 ng/mL identified (AUC = 0.73, p = 0.0259). Importantly, these low values lie below the therapeutic exposure range. Clinically, the interpretation of such low concentrations also depends on sampling time: values below this threshold at expected peak times almost certainly indicate underexposure, whereas trough concentrations between 1 and 4 μg/mL remain within an acceptable and safe range [10]. In these scenarios, minor deviations introduced by conversion formulas are unlikely to alter clinical decisions. Overall, integrating the analytical findings with clinical considerations suggests that these conversion approaches are feasible for TDM when applied within appropriate concentration ranges and clinical contexts.

For clinical application, the VAMS-based approach enabled more comprehensive PK profiling in lung-transplant recipients than conventional venipuncture could feasibly provide. The eight-point VAMS sampling captured secondary peaks attributable to enterohepatic recirculation, yielding complete AUC estimates near the lower therapeutic threshold of 30 mg·h/L [10–12]. Despite these subtherapeutic AUCs, both cases exhibited leukopenia, most likely due to the combined myelotoxic effects of concomitant immunosuppressants and anti-infective agents, including calcineurin inhibitors, sulfamethoxazole–trimethoprim, and valganciclovir [3, 40, 41]. This observation suggests that myelosuppression may result from cumulative pharmacodynamic interactions rather than excessive MPA exposure. Therefore, a VAMS-based full-profile approach can assist in distinguishing PK underexposure from pharmacodynamic toxicity and guide individualized dose adjustment in complex transplant regimens. Moreover, the observed enterohepatic recirculation—manifested as a secondary MPA peak at approximately 6–8 h post-dose—further supports the clinical value of dense sampling [42, 43]. This character highlights the advantage of using VAMS as the sampling strategy for exposure assessment. Capturing these delayed peaks is crucial for accurate AUC estimation, as neglecting them can lead to substantial underestimation of systemic exposure. VAMS, by enabling convenient and minimally invasive multi-time-point sampling, facilitates a more complete PK evaluation without increasing patient burden.

Moreover, TAC concentrations measured from whole blood showed good agreement with those from VAMS samples. By reducing the need for venous blood sampling and enabling more frequent monitoring, VAMS can enhance individualized immunosuppressive therapy, while also improving patient adherence and comfort.

Although eight-point sampling provides a comprehensive PK profile and serves as a robust reference for AUC determination, such an intensive approach is unlikely to be practical for routine clinical monitoring. The primary purpose of employing an 8-point design in this study was to generate high-resolution exposure data that can inform the development of simplified monitoring strategies. Importantly, this intensive dataset establishes a PK reference framework that enables subsequent model-informed analyses, including population PK modeling and the derivation of LLS. These approaches may allow accurate prediction of full AUC using substantially fewer sampling time points, thereby improving clinical feasibility while preserving precision. Ultimately, reducing the number of required finger-pricks is critical to enhancing patient acceptability and facilitating the integration of VAMS-based monitoring into clinical practice.

### Limitations

Despite these promising results, several limitations should be acknowledged. First, our study had a relatively small sample size (n = 21), which may restrict the generalizability of the findings. Second, the patient population primarily consisted of kidney and lung transplant recipients, limiting extrapolation to other transplant populations who might also benefit from VAMS-based MPA monitoring. Third, all VAMS sampling and handling were performed by healthcare professionals, which may not fully reflect real-world scenarios where patients collect samples themselves. An additional limitation is that VAMS specimens were generated from venous whole blood rather than finger-stick capillary samples, which does not fully replicate real-world sampling conditions; however, published data suggest negligible capillary–venous bias for MPA and other immunosuppressants, indicating limited impact on clinical applicability [27, 34]. Finally, the overall Hct values of our patients were significantly lower than those of the general population, raising the need to further evaluate the applicability of these findings in individuals with normal Hct ranges. Nevertheless, because the VAMS samples in this study were collected from real transplant patients, this phenomenon further highlights the importance of carefully considering Hct effects, particularly in patients with low Hct values, and of selecting an appropriate concentration conversion strategy for this patient population.

### Conclusion

In summary, this study demonstrates that VAMS sampling, combined with appropriate Hct-adjusted conversion, can provide reasonable and clinically interpretable MPA and TAC measurements. The method shows strong potential for conditional integration into routine transplant TDM. Future work should evaluate home-based self-sampling, validate LSS derived from VAMS, and explore broader applicability across diverse transplant populations.

## Data Availability

The raw data supporting the conclusions of this article will be made available by the authors, without undue reservation.
