# Supplementary material for: Evaluation of hematocrit-adjusted conversion strategies for mycophenolic acid and tacrolimus monitoring using volumetric absorptive microsampling in lung and renal transplant recipients
Source: J Pharm Pharm Sci. 2026 Mar 26;29:16123. doi: 10.3389/jpps.2026.16123 (PMC13061867; doi:10.3389/jpps.2026.16123)
Supplement: Supplementary file 1 [file Supplementaryfile1.docx]

**SUPPLEMENTAL MATERIALS**

Figure S1. Multiple reaction monitoring (MRM) data and retention times for the plasma quantification of

MPA and TAC, along with their isotopic internal standards, under optimized chromatographic conditions.


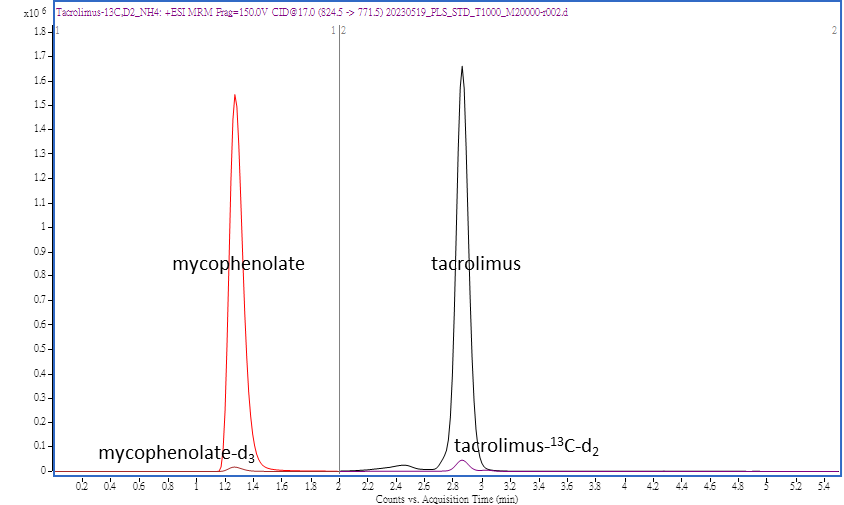


Figure S2. ROC curve analysis evaluating the predictive performance of combined results (individualized formula A, B, C, and D) for meeting the analytical requirement of ±10% mean difference.


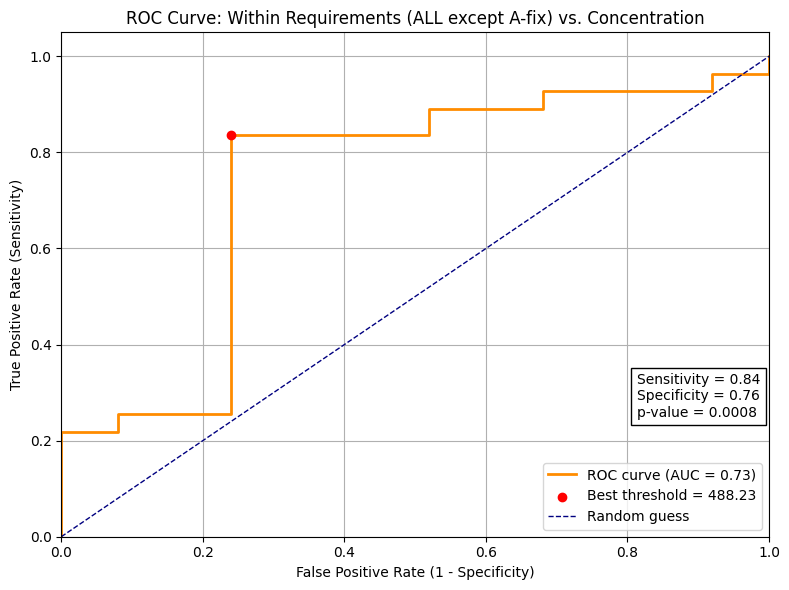


| Table S1. Mass spectrometer parameters | | | |
| --- | --- | --- | --- |
| Compound | *m*/*z* | MRM | CE |
| Mycophenolate | [M+NH_4_]^+^ | 321.1→207.1 | 17 |
| Mycophenolate-d_3_ | [M+NH_4_]^+^ | 324.2→210.1 | 17 |
| Tacrolimus | [M+H]^+^ | 821.5→768.4 | 17 |
| Tacrolimus-^13^C-d_2_ | [M+H]^+^ | 824.5→771.5 | 17 |
| CE: collision energy; MRM: multiple reaction monitoring. | | | |

| Table S2. Linearity of different matrix | | | |
| --- | --- | --- | --- |
| Compound | Types of Sampling | Linear range (ng/mL) | R^2^ |
| MPA | plasma | 10-20000 | 0.996 |
| TAC | blood | 0.5-500 | 0.995 |
| MPA | VAMS | 10-20000 | 0.997 |
| TAC | VAMS | 0.5-500 | 0.995 |
| MPA: mycophenolic acid; TAC: tacrolimus; VAMS: volumetric absorptivce microsampling. | | | |

| Table S3. Precision and accuracy for quantification of plasma MPA and whole blood TAC. | | | | | | | | |
| --- | --- | --- | --- | --- | --- | --- | --- | --- |
| Compound name | Sample Type | Spike concentration (ng/mL) | Intra-day(n=3) | | Extraction recovery | | Matrix effect | |
|  |  |  | Accuracy  (%) | Precision (RSD%) | Recovery  (%) | Precision (RSD%) | Matrix effect (%) | Precision (RSD%) |
| MPA^a^ | Plasma | LLOQ | 82.25±1.44 | 1.76 | NA | NA | NA | NA |
|  |  | LQC | 96.11±5.01 | 5.21 | 84.97±9.17 | 10.79 | 71.64±3.10 | 4.34 |
|  |  | MQC | 103.01±1.58 | 1.53 | 94.15±0.56 | 0.60 | 76.39±1.05 | 1.37 |
|  |  | HQC | 97.70±2.39 | 2.45 | 98.81±0.20 | 0.20 | 64.70±0.81 | 1.26 |
| TAC^b^ | Blood | LLOQ | 85.94±5.08 | 5.91 | NA | NA | NA | NA |
|  |  | LQC | 89.36±5.29 | 5.92 | 96.86±2.27 | 2.34 | 76.47±2.24 | 2.93 |
|  |  | MQC | 100.95±3.40 | 3.36 | 95.20±3.90 | 4.09 | 80.58±6.02 | 7.48 |
|  |  | HQC | 94.90±4.56 | 4.81 | 87.55±7.61 | 8.70 | 69.23±2.88 | 7.70 |
| HQC: higher quality control; LLOQ: lower limit of quantification; LQC: lower quality control; MPA: mycophenolic acid; MQC: middle quality control; RSD: relative standard deviation; TAC: tacrolimus.  ^a^LLOQ and LQC, MQC, and HQC concentrations of samples for MPA were 10, 20, 200, and 2,000 ng/mL respectively.  ^b^LLOQ and LQC, MQC, and HQC concentrations of samples for TAC were 0.5, 1, 10, and 100 ng/mL respectively. | | | | | | | | |

| Table S4. Patient characteristics | |
| --- | --- |
| Characteristics | Total of 21 patients ^a^ |
| Age (y/o) | 47.5 (44-59) |
| Gender | Male: 15 (17.4%) |
| Body Height (cm) | 168 (161-170) |
| Body Weight (kg) | 62 (54.3-68.25) |
| Organ Transplantation | Lung: 10  Renal: 11 |
| MPA Brand | Cellcept® (Mycophenolate Mofetil): 10 Myfortic® (Mycophenolate Sodium): 11 |
| Hct (%) | 29 (27-33) |
| MPA VAMS conc. (ng/mL) | 483.62 (143.15-916.56) |
| MPA Plasma conc. (ng/mL) | 725.41 (210.07-1261.54) |
| TAC VAMS conc. (ng/mL) | 4.76 (3.71-32.58) |
| TAC Whole Blood conc. (ng/mL) | 6.00 (4.70-8.00) |
| Note: All patients received Prograf® (immediate-release TAC) except for two patients, who received Advagraf® (prolonged-release TAC).  Hct: hematocrit; MPA: mycophenolic acid; TAC: tacrolimus; VAMS: volumetric absorptive micosampling.  ^a^ Median value (IQR) | |
